# Atypical GATA transcription factor TRPS1 represses gene expression by recruiting CHD4/NuRD(MTA2) and suppresses cell migration and invasion by repressing *TP63* expression

**DOI:** 10.1038/s41389-018-0108-9

**Published:** 2018-12-19

**Authors:** Yuzhi Wang, Xue Lin, Xue Gong, Lele Wu, Jun Zhang, Weiguang Liu, Jian Li, Liming Chen

**Affiliations:** 1 Jiangsu Key Laboratory for Molecular and Medical Biotechnology, College of life Science, Nanjing Normal University, 210023 Nanjing, P. R. China; 2 Institute of Life Science, Southeast University, 210096 Nanjing, P. R. China; 3 Department of Bioinformatics, School of Biomedical Engineering and Informatics, Nanjing Medical University, 210029 Nanjing, P. R. China

## Abstract

Transcriptional repressor GATA binding 1 (TRPS1), an atypical GATA transcription factor, functions as a transcriptional repressor and is also implicated in human cancers. However, the underlying mechanism of TRPS1 contributing to malignancy remains obscure. In the current study, we report that TRPS1 recognizes both gene proximal and distal transcription start site (TSS) sequences to repress gene expression. Co-IP mass spectrometry and biochemical studies showed that TRPS1 binds to CHD4/NuRD(MTA2). Genome-wide and molecular studies revealed that CHD4/NuRD(MTA2) is required for TRPS1 transcriptional repression. Mechanically, TRPS1 and CHD4/NuRD(MTA2) form precision-guided transcriptional repression machinery in which TRPS1 guides the machinery to specific target sites by recognizing GATA elements, and CHD4/NuRD(MTA2) represses the transcription of target genes. Furthermore, *TP63* was identified and validated to be a direct target of TRPS1-CHD4/NuRD(MTA2) complex, which represses *TP63* expression by involving decommission of *TP63* enhancer in the described precision-guided manner, leading to a reduction of the ΔNp63 level and contributing to migration and invasion of cancer cells.

## Introduction

Transcriptional repressor GATA binding 1 (TRPS1), an atypical member of the family of GATA transcriptional factors, has been characterized as the first example of a GATA protein with intrinsic transcriptional repression activity for >15 years^1^. How TRPS1 functions as a transcriptional repressor is largely unknown.

Mutations in TRPS1 were documented to be an underlying cause of Tricho-rhino-phalangeal syndrome type I, an autosomal-dominant disorder characterized by craniofacial and skeletal malformations^2^. TRPS1 was also implicated in cancer as its elevated expression was observed in various human malignancies, including osteosarcoma^3^, colon cancer^4^, and breast cancer^5^. By using in vivo transposon-based forward genetic screening aimed at screening breast cancer-driver genes, we^6^ and others^7^ recently identified TRPS1 as a driver gene in breast cancer indicating its role in breast cancer pathogenesis. However, how TRPS1 contributes to breast cancer is still obscure.

CHD (chromodomain helicase DNA-binding protein) proteins are important regulators of transcription and can be divided into three subfamilies: CHD1-CHD2, CHD3-CHD4, and CHD5-CHD9^8^. The CHD3-CHD4 subfamily, which lacks a DNA-binding domain, includes CHD3 (also known as Mi-2α) and CHD4 (also known as Mi-2β), which are the central components of the nucleosome-remodeling and histone deacetylase (NuRD, also known as Mi-2) complex^9^, which mainly functions in transcriptional repression^10^. CHD4 is an essential subunit for the transcriptional repressive function of NuRD complex, and multiple domains of CHD4 are required for transcriptional repression by NuRD complexes^11^.

p63 is a member of the p53 family^12^, which is commonly mutated in human cancers and is well documented as an important tumor-suppressor gene^13^. Unlike p53, owing to the complexity of the *TP63* gene, the role of p63 in cancer is still controversial and an area of intense research. *TP63* encodes multiple p63 isoforms^14^ that can be placed in two categories: TAp63 and ΔNp63, which are with and without an N-terminal transactivation domain, respectively. Experimental evidence suggests that TAp63 acts as a tumor suppressor, whereas ΔNp63 has oncogenic functions^15,16^. ΔNp63 has been suggested to be an important driver for promoting breast tumor progression and metastasis by transcriptional activation of MTSS1^17^ or PI3K/CD44v6 axis^18^. However, transcriptional regulation of *TP63* in breast cancer is still not fully understood.

In this study, we addressed how TRPS1 functions as a transcriptional repressor and contributes to breast cancer pathogenesis. Our results illustrated that TRPS1 repressed gene expression by forming a transcriptional repression complex, TRPS1-CHD4/NuRD(MTA2), and directed the complex to transcriptional regulatory regions in a precision-guided manner. We further identified that the *TP63* gene was repressed by this precision-guided machinery of TRPS1-CHD4/NuRD(MTA2) complex, which decommissions its enhancer leading to a decrease in ΔNp63 and enhancing the metastatic ability of breast cancer cells.

## Results

### Identification of genome-wide transcription targets of TRPS1

**Fig. 1:**
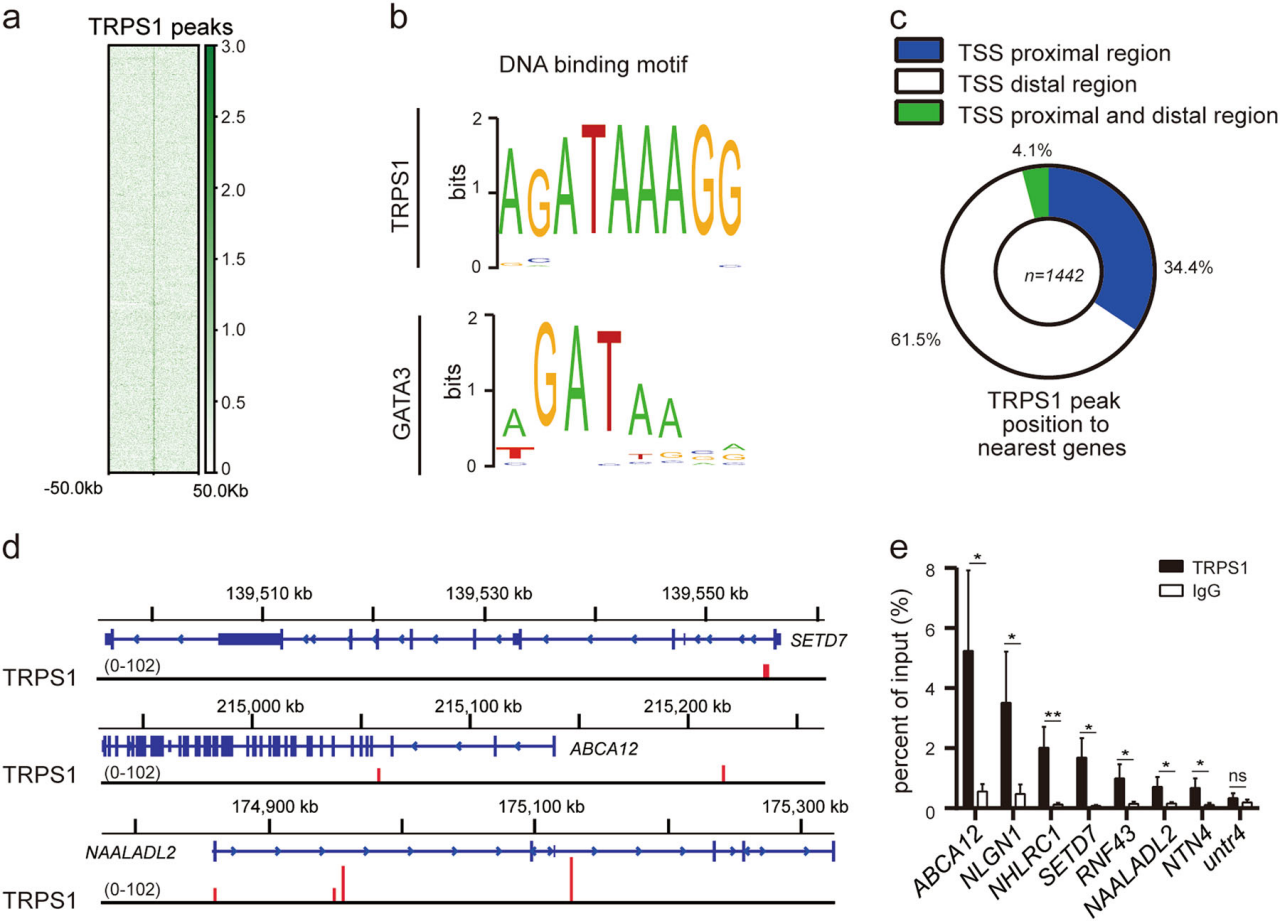
Genome-wide transcription target analysis for TRPS1. **a** ChIP-seq density map of TRPS1-binding sites. **b** TRPS1- and GATA3-binding motif analysis. GATA3 motif analysis was based on data from JASPAR MA0037.3^39^. **c** Genomic distribution of TRPS1 determined by ChIP-seq analysis. **d** Genome browser track examples of the binding of TRPS1 on representative target genes, *SETD7*, *ABCA12*, and *NAALADL2*. **e** ChIP-qPCR analysis of TRPS1 candidate target genes: *ABCA12*, *NLGN1*, *NHLRC1*, *SETD7*, *RNF43*, *NAALADL2*, and *NTN4*. Untr4 is a well-characterized untranscribed genomic region and used as a comparative control. Each bar represents the mean ± SD for triplicate experiments. The *t* test was used for calculation of statistical significance. *p < 0.05, **p < 0.01

Although, like other GATA transcription factors, TRPS1 is believed to recognize the WGATAR consensus binding sequence, there are no investigations for genomic binding profiles of TRPS1. To address this, we carried out chromatin immunoprecipitation–sequencing (ChIP-seq) analysis in T47D cells with antibodies against TRPS1 (Fig. 1a and the peaks shown in Supplementary Table 1). The binding motif analysis showed that TRPS1 recognized AGATAAGG elements containing WGATAR, indicating that TRPS1, as an atypical GATA transcription factor, binds to consensus GATA-binding elements and might exhibit different binding preference compared to typical GATA factors, such as GATA-3 (Fig. 1b).To further explore the regulatory context of TRPS1, we divided the TRPS1 enrichment regions into transcription start site (TSS) proximal (peaks enriched within 5 kb from TSS sites) and distal regions (peaks enriched outside 5 kb from TSS sites). The results revealed that TRPS1 could bind to both gene proximal and distal TSS regions (Fig. 1a–c). As is shown in Fig. 1c, 61.5%, 34.4%, and 4.1% of TRPS1 enrichment peaks were present in TSS distal, proximal, and both proximal and distal regions, respectively. For example, TRPS1 enrichment was observed in the TSS distal region of *ABCA12* gene, TSS proximal region of *SETD7* gene, and both TSS proximal and distal regions of *NAALADL2* gene (Fig. 1d). The chromatin immunoprecipitation–quantitative polymerase chain reaction (ChIP-qPCR) analysis confirmed the enrichment of TRPS1 in the corresponding regions of *ABCA12*, *SETD7*, and *NAALADL2* as well as other selected genes, including *NLGN1*, *NHLRC1*, and *RNF43* using *untr4*, a well-characterized untranscribed genomic region, as a control for the comparative analysis (Fig. 1e).

### TRPS1 binds CHD4/NuRD(MTA2) to form TRPS1-CHD4/NuRD(MTA2) complex

**Fig. 2:**
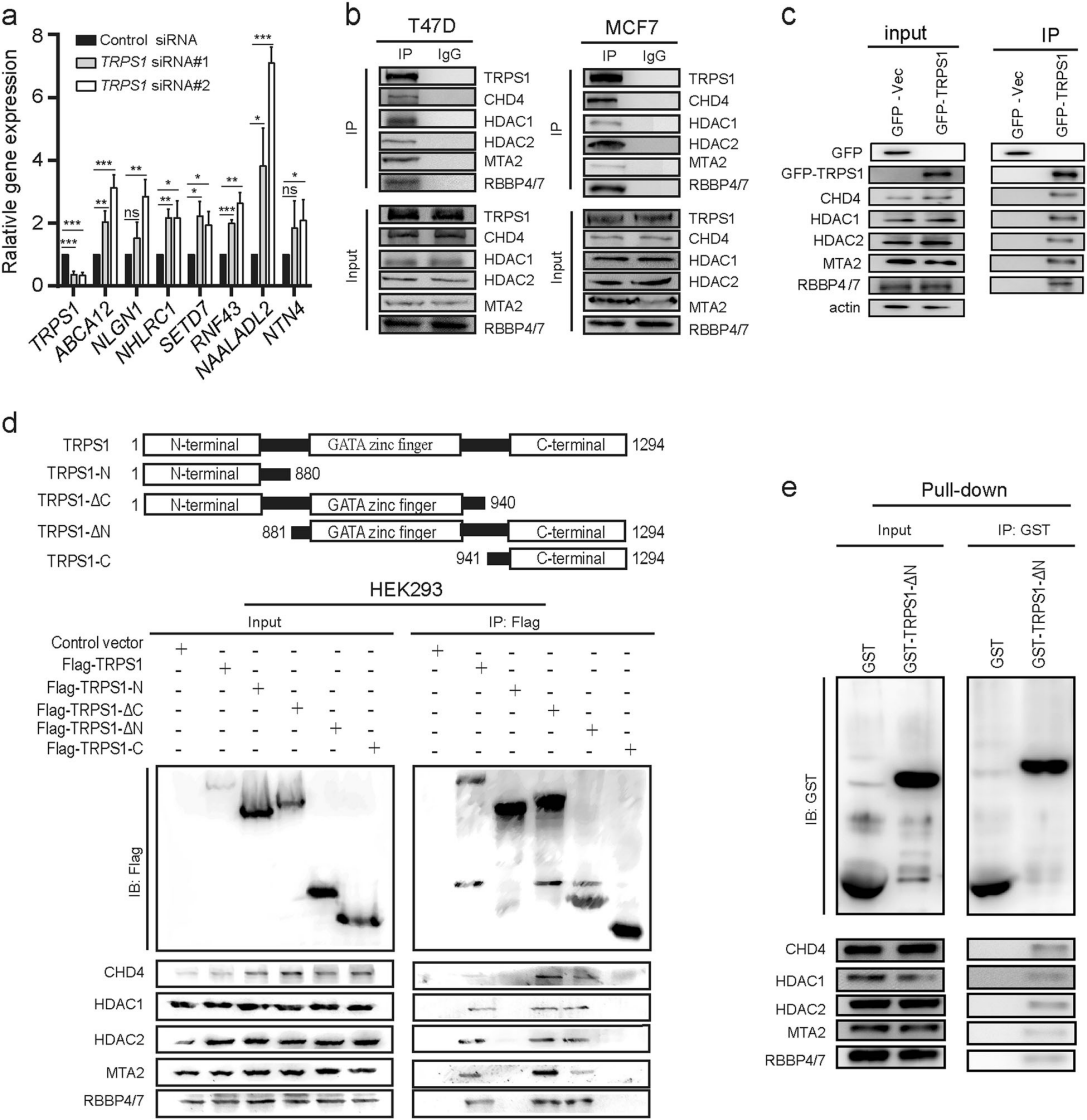
TRPS1 interacts with CHD4/NuRD(MTA2) complex. **a** RT-qPCR validation of *ABCA12*, *NLGN1*, *NHLRC1*, *SETD7*, *RNF43*, *NAALADL2*, and *NTN4* expression upon silencing TRPS1. *t* Test was used for calculation of statistical significance. *p < 0.05, **p < 0.01, ***p < 0.001. **b** TRPS1 interacts with major components of CHD4/NuRD(MTA2) complex: CHD4, HDAC1, HDAC2, MTA2, and RBBP4/7 in T47D and MCF7 cells. **c** Ectopically overexpressed TRPS1 in MDA-MB-231 cells interacts with major components of CHD4/NuRD(MTA2) complex: CHD4, HDAC1, HDAC2, MTA2, and RBBP4/7. **d** Co-IP analysis of the interaction between ectopically overexpressed Flag-tagged full-length TRPS1 or truncated TRPS1 proteins and major components of CHD4/NuRD(MTA2) complex: CHD4, HDAC1, HDAC2, MTA2, and RBBP4/7. The schematic diagram of TRPS1 truncates deciphered in the upper panel. **e** GST pull-down assays TRPS1-ΔN and whole-cell lysates of HEK293T cells

To further characterize the transcription output profiles mediated by TRPS1, we performed RNA-seq analysis in T47D cells with or without silencing *TRPS1*. Consistent with the current notion that TRPS1 functions as a transcriptional repressor, *ABCA12*, *NLGN1*, *NHLRC1*, *SETD7*, *RNF43*, *NAALADL2*, and *NTN4* genes, with TRPS1 enrichment at their regulatory regions according to the ChIP-seq and ChIP-qPCR analysis, showed upregulated transcription upon silencing *TRPS1*. This was evident by the >2-fold change (*p* < 0.05) observed in RNA-seq data and confirmed by reverse transcription qPCR (RT-qPCR; Fig. 2a and Supplementary Table 2). In RNA-seq data, upon silencing *TPRS1*, 476 genes and 550 genes were upregulated or downregulated (fold change >2, *p* < 0.05), respectively (Supplementary Table 2). We noticed that not all genes with TRPS1 enrichment at their regulatory elements showed upregulation upon silencing *TRPS1* (476 upregulated genes upon silencing *TRPS1*, whereas 1442 genes with TRPS1 enrichment). We hypothesized that TRPS1 might recruit other regulatory factors to form a functional machinery to repress gene expression. Upon re-analysis of the TRPS1 interactome reported by our group previously, we found that CHD3/4, HDAC1/2, MTA2, GATA2A/B, and RBBP4/7, which are core components of the NuRD complex^19^, were included in the TRPS1 interactome list^20^ (Supplementary Fig. 1). This result indicated that TRPS1 recruits CHD4/NuRD/MTA2 complex to exert its transcription repressor function. To validate this point, we first confirmed the physical interaction between TRPS1 and CHD4/NuRD/MTA2 complex using co-immunoprecipitation (Co-IP). The results showed that CHD4, HDAC1/2, MTA2, and RBBP4/7 could be co-purified with TRPS1 in the Co-IP assay using TRPS1 antibodies in both T47D and MCF7 cells with high endogenous TRPS1 expression (Fig. 2b). Similar results were observed in MDA-MB-231 cells with undetectable endogenous TRPS1 expression but with ectopically overexpressed TRPS1 (Fig. 2c). To further verify which domain of TRPS1 was responsible for interacting with CHD4/NuRD/MTA2 complex, we carried out Co-IP in HEK293T cells with ectopic overexpression of TRPS1 or serial TRPS1 domain truncates. CHD4, HDAC1/2, MTA2, and RBBP4/7 could be co-immunoprecipitated with either full-length TRPS1 or TRPS1-truncated mutants that contained GATA-binding domain coupled with either N-terminal domain or C-terminal domain but could not be co-immunoprecipitated with TRPS1 truncates containing the only N-terminal domain or C-terminal domain of TRPS1 (Fig. 2d). To further confirm this observation, we carried out pull-down assays. We constructed plasmids containing full-length TRPS1 and its serial truncates for generating recombinant proteins of TRPS1 and its truncates in the bacterial system. Only recombinant TRPS1-ΔN truncate proteins could be purified as soluble recombinant proteins. Consistent with the Co-IP experiments described above, pull-down assay confirmed that purified recombinant TRPS1-ΔN protein was able to pull down CHD4, HDAC1/2, MTA2, and RBBP4/7 (Fig. 2e). These results suggested that TRPS1 physically interacted with CHD4/NuRD(MTA2) and GATA-binding domains and the C-terminal domain of TRPS1 was sufficient for forming TRPS1-CHD4/NuRD(MTA2) complex.

### TRPS1 nucleates CHD4/NuRD(MTA2) to form precision-guided machinery to repress target gene expression

**Fig. 3:**
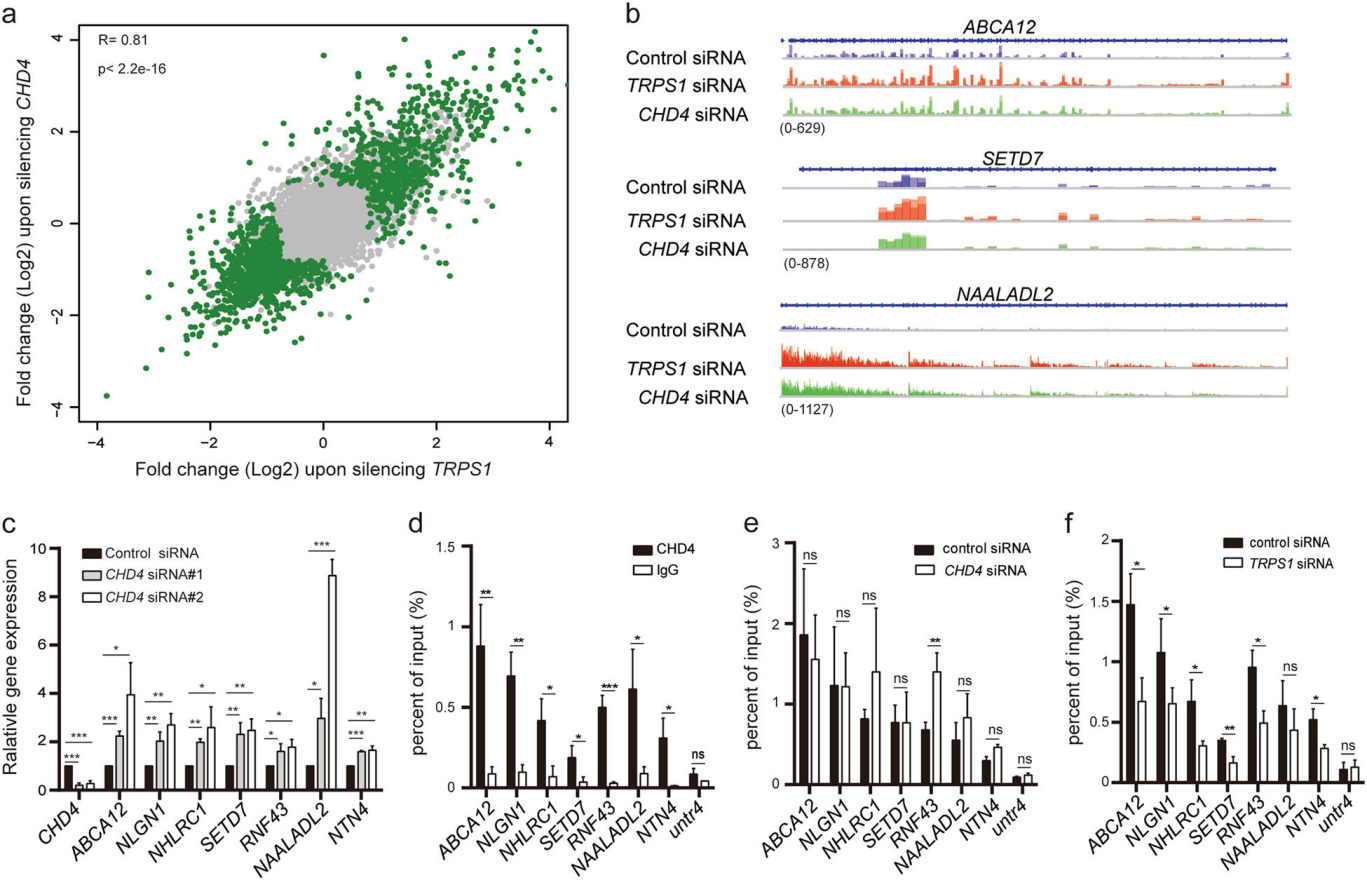
TRPS1 nucleates CHD4/NuRD(MTA2) complex to repress target gene expression. **a** Scatter plots demonstrating the correlation between the transcriptional outputs mediated by TRPS1 and CHD4. Genes indicate significantly differentially expressed genes. Correlation coefficients were calculated using Pearson correlation coefficient and the *p* value was calculated using *t* test (*p* < 2.2e−16). **b** RNA-seq track examples of the selected genes, *SETD7*, *ABCA12*, and *NAALADL2*. **c** RT-qPCR validation of *ABCA12*, *NLGN1*, *NHLRC1*, *SETD7*, *RNF43*, *NAALADL2*, and *NTN4* expression upon silencing *CHD4*. **d** ChIP-qPCR analysis of CHD4 enrichment on regulatory regions of *ABCA12*, *NLGN1*, *NHLRC1*, *SETD7*, *RNF43*, *NAALADL2*, and *NTN4*. **e** ChIP-qPCR analysis of TPRS1 enrichment on regulatory regions of *ABCA12*, *NLGN1*, *NHLRC1*, *SETD7*, *RNF43*, *NAALADL2*, and *NTN4* upon silencing *CHD4*. **f** ChIP-qPCR analysis of CHD4 enrichment on regulatory regions of *ABCA12*, *NLGN1*, *NHLRC1*, *SETD7*, *RNF43*, *NAALADL2*, and *NTN4* upon silencing *TRPS1*. Each bar represents the mean ± SD for triplicate experiments. *t* Test was used for statistical significance analysis. *p < 0.05, **p < 0.01, ***p < 0.001. **d**–**f**: *Untr4* acts as a comparative control, and each bar represents the mean ± SD for triplicate experiments

We investigated whether CHD4/NuRD(MTA2) was required as an essential and functional component of the TRPS1 transcriptional repression complex. We first silenced *CHD4*, an essential component of CHD4/NuRD(MTA2), and analyzed the transcription output mediated by this dysfunctional complex by RNA-seq and compared it with that without silencing *CHD4*. The correlation analysis between TRPS1- and CHD4-mediated transcription output exhibits a high *R*-value (*R* = 0.81, *p* < 2.2e−16), suggesting that TRPS1 and CHD4/NuRD(MTA2) were functionally linked in determining the transcriptional outputs in our investigating system (Fig. 3a, b, Supplementary Fig. 2 and Supplementary Table 3). Consistent with silencing *TRPS1*, transcriptional upregulation of *ABCA12*, *NLGN1*, *NHLRC1*, *SETD7*, *RNF43*, *NAALADL2*, and *NTN4* was observed when *CHD4* was silenced and could be verified by RNA-seq and RT-qPCR (Fig. 3c and Supplementary Table 3).

As described above, TRPS1 binds to specific DNA sequence (Fig. 1) and either depletion of TRPS1 or dysfunction of CHD4/NuRD(MTA2) by depleting its essential component, CHD4, resulted in the loss of transcriptional repression of a set of target genes. We hypothesized that TRPS1 nucleates CHD4/NuRD(MTA2) complex to form precision-guided machinery, which precisely represses the expression of target genes. In this complex, TRPS1 functions as the guiding element by targeting specific DNA sequences in the genome and CHD4/NuRD(MTA2) represses expression of the target genes. We performed ChIP-qPCR using CHD4 antibodies and observed enrichment of CHD4 at the regulatory regions of *ABCA12*, *NLGN1*, *NHLRC1*, *SETD7*, *RNF43*, *NAALADL2*, and *NTN4* together with TRPS1 enrichment (Fig. 3d). Furthermore, silencing *CHD4* did not affect TRPS1 enrichment at the regulatory regions of the investigated genes, while silencing *TRPS1* led to the loss of enrichment of CHD4 at these regions (Fig. 3e, f). Taken together, these results indicated that TRPS1 nucleated CHD4/NuRD(MTA2) complex to repress target gene expression.

### TRPS1-CHD4/NuRD(MTA2) machinery represses *TP63* expression by involving decommission of its enhancer

**Fig. 4:**
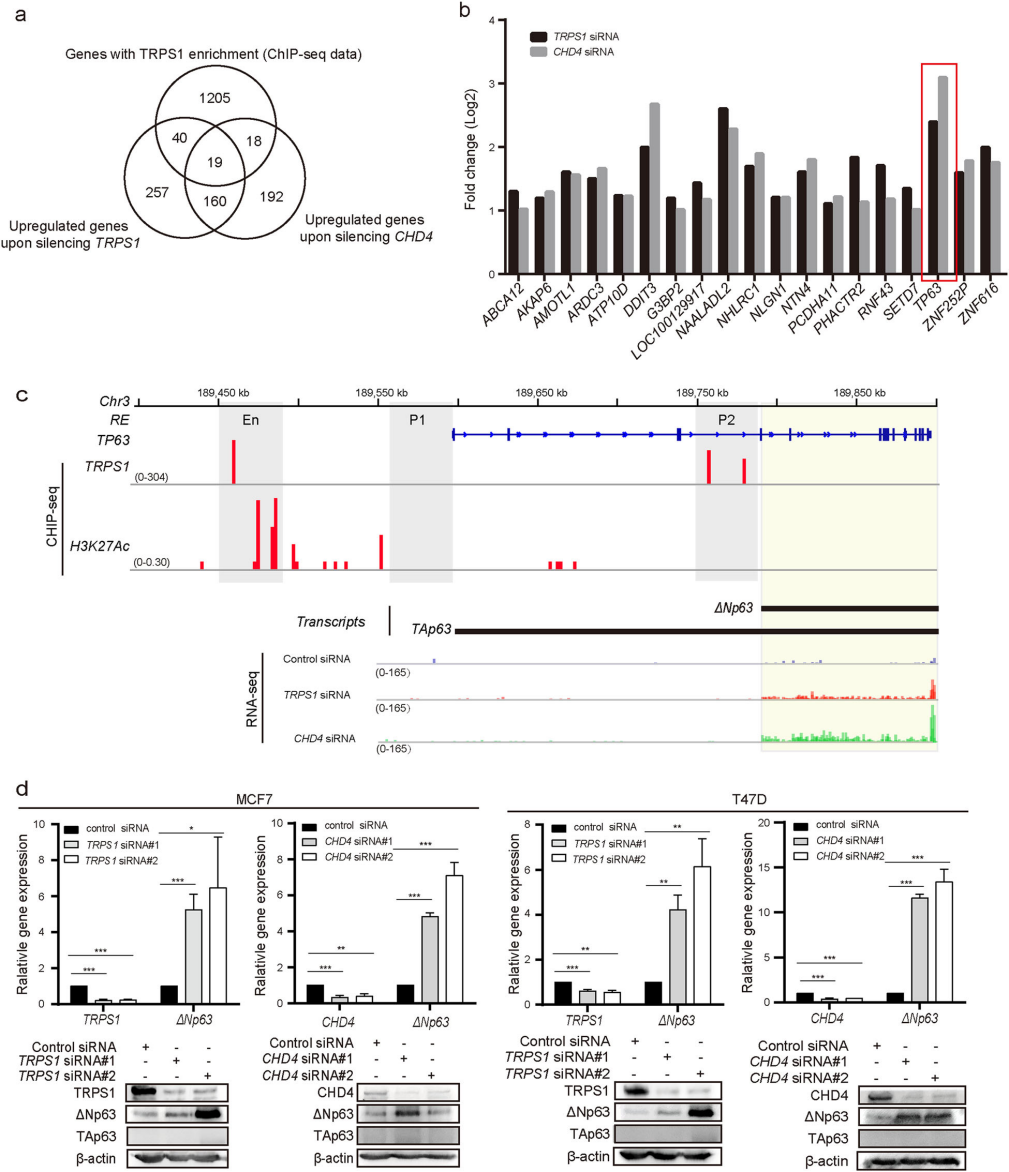
TRPS1-CHD4/NuRD(MTA2) regulates ΔNp63 expression by targeting regulatory region of *TP63*. **a** Venn diagram displays overlapping gene analysis of TRPS1 ChIP-seq-enriched genes and RNA-seq data of upregulated genes after the TRPS1and CHD4 knockdown. **b** Histogram of upregulated TRPS1 enrichment genes after TRPS1 and CHD4 knockdown. **c** ChIP-seq track of TRPS1 occupancy in the *TP63* regulatory region and RNA-seq track of the *TP63* gene expression. En, P1, and P2 indicate the enhancer, P1 promoter, and P2 promoter region of *TP63*, respectively. **d** ΔNp63 was upregulated at the mRNA and protein levels upon silencing *TRPS1* or *CHD4*. Each bar represents the mean ± SD for triplicate experiments. *t* Test was used for statistical significance calculation. *p < 0.05, **p < 0.01, ***p < 0.001

We attempted to identify a specific gene targeted and transcriptionally repressed by TRPS1-CHD4/NuRD(MTA2) complex. For this purpose, *TP63* was selected as it showed consistent upregulation with the largest fold increase upon silencing *TRPS1* or *CHD4* (Fig. 4a, b). *TP63* encodes multiple p63 proteins including isoforms of two main groups: those with a full-length transactivation domain (referred to as the TAp63) and those lacking the transactivation domain (ΔNp63)^21^. From RNA-seq data, we found that only expression of isoforms coding ΔNp63 was detectable and showed upregulation upon silencing TRPS1 or CHD4 (Fig. 4c). By using RT-qPCR and western blotting, we confirmed that ΔNp63 was upregulated at the mRNA and protein levels, respectively, upon silencing either *TRPS1* or *CHD4*, while TAp63 protein was not detected in any of the investigated samples (Fig. 4d). These results suggested that TRPS1-CHD4/NuRD(MTA2) machinery repressed the expression of *TP63*, leading to decreased expression of ΔNp63.

**Fig. 5:**
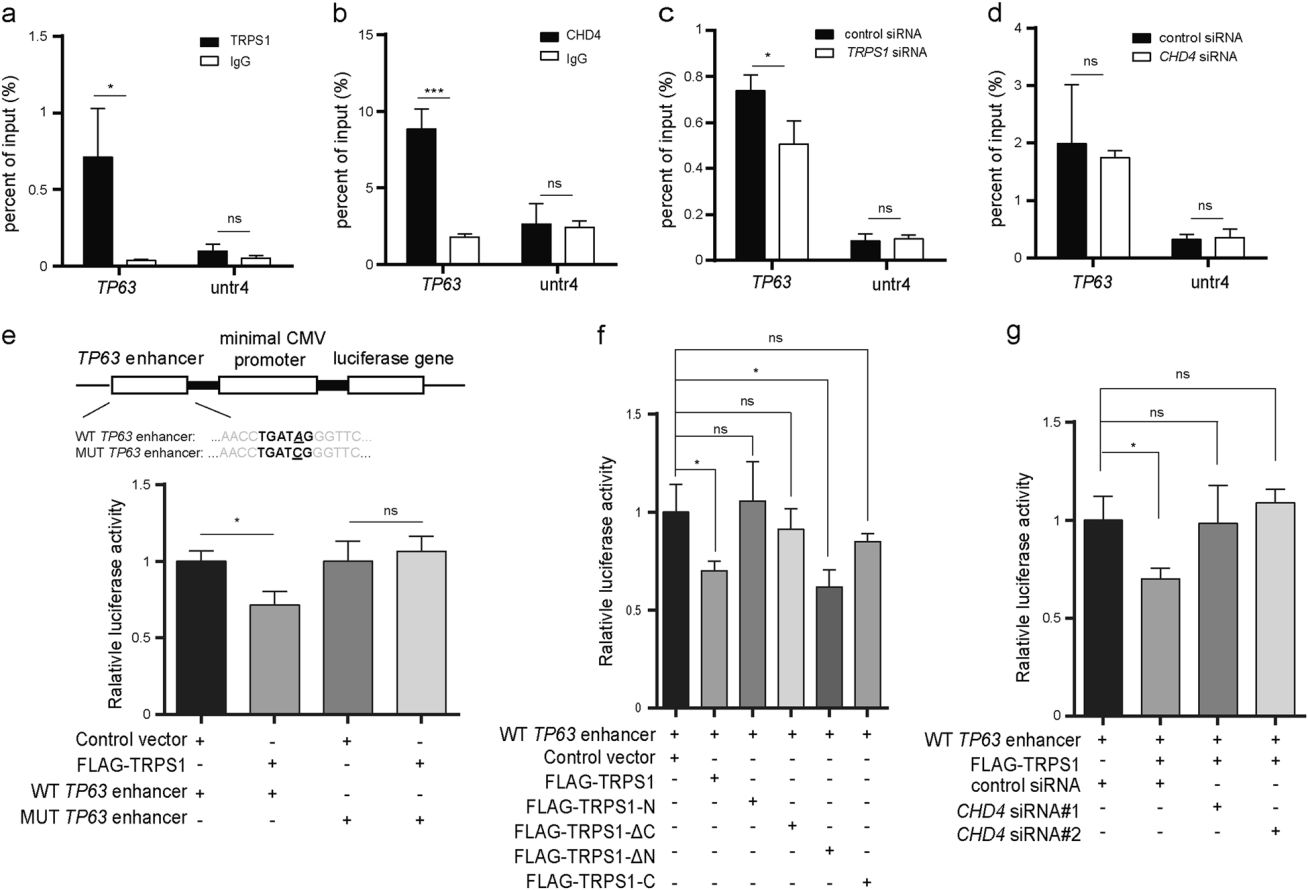
TRPS1-CHD4/NuRD(MTA2) decommissions *TP63* enhancer in the precision-guided manner. **a** ChIP-qPCR confirmed enrichment of TRPS1 at *TP63* enhancer. **b** ChIP-qPCR confirmed enrichment of CHD4 at *TP63* enhancer. **c** Enrichment of CHD4 at *TP63* enhancer was reduced upon silencing *TRPS1* by ChIP-qPCR. **d** Enrichment of TRPS1 at *TP63* enhancer was not affected upon silencing *CHD4* by ChIP-qPCR. **a**–**d** Each bar represents the mean ± SD for triplicate experiments. **e** *TP63* enhancer luciferase assay with ectopic overexpression of TRPS1 in HEK293T cells. **f** *TP63* enhancer luciferase assay with ectopic overexpression of TRPS1 and its truncates in HEK293T cells. **g** *TP63* enhancer luciferase assay with or without silencing *CHD4* in HEK293T cells with ectopic overexpression of TRPS1. **e**–**g**: Each bar represents the mean ± SD for triplicate experiments. *t* Test was used for calculation of statistical significance. *p < 0.05, ***p < 0.001

TAp63 and ΔNp63 are reported to be regulated by the P1 and P2 promoters, respectively. ChIP-Seq data showed that TRPS1 enriched at both P2 and an additional region at upstream of P1 but not at P1. Regions with H3K27ac enrichment were suggested to be candidate enhancers^22^. Via retrieving and analyzing H3K27ac ChIP-seq data for T47D (GSM1693025), we found that the additional region at upstream of P1 but not P1 and P2 shows enrichment of H3K27ac, suggesting that the additional region at upstream of P1 is an enhancer and TRPS1 was likely to repress *TP63* expression by targeting both enhancer and P2 promoter of *TP63* (Fig. 4c). Since regulation of ΔNp63 expression by the P2 promoter is well documented, we focused on its regulation by the *TP63* enhancer element. To test that TRPS1-CHD4/NuRD(MTA2) machinery repressed ΔNp63 expression by directly targeting its enhancer, we performed ChIP-qPCR and detected ~7-fold enrichment of TRPS1 and ~5-fold enrichment of CHD4 at the *TP63* enhancer. These observations indicated that the TRPS1-CHD4/NuRD(MTA2) machinery directly targeted *TP63* enhancer (Fig. 5a, b). When ChIP-qPCR was performed using CHD4 antibody with or without *TRPS1* silencing, CHD4 enrichment at *TP63* enhancer was reduced upon silencing *TRPS1* (Fig. 5c). In comparison, upon silencing *CHD4*, TRPS1 enrichment at *TP63* enhancer remained unaffected (Fig. 5d).

To further confirm that *TP63* enhancer is functional enhancer and contributes to repression of *TP63* expression by TRPS1-CHD4/NuRD(MTA2) complex, we carried out enhancer trap analysis via cloning *TP63* enhancer sequence in pGL3-basic coupled with minimal cytomegalovirus (CMV) promoter driving the luciferase gene followed by luciferase reporter assay. The results showed that the luciferase activity of *TP63* enhancer was significantly attenuated upon overexpression of *TRPS1*. On the other hand, TPRS1 was unable to attenuate luciferase activity when *TP63* enhancer was mutated from GATA to GATC in the central GATA-binding sequence (Fig. 5e). These results indicated that TRPS1 was able to repress transcriptional activation of *TP63* by recognizing the GATA sequence in *TP63* enhancer. We further investigated which domain of TRPS1 was required for enhancer repression by overexpressing full-length TRPS1 and a series of TRPS1 truncates. As described above, TRPS1-ΔN, containing GATA domain and C-terminal domain of TRPS1, was sufficient to bind CHD4/NuRD(MTA2) complex and reduced transcription of *TP63* enhancer causing a significant decrease in luciferase activity comparable to that of full-length TRPS1, indicating that C-terminal region of TRPS1 is a repression domain (Figs. 2 and 5f). Furthermore, overexpression of TRPS1 was unable to inhibit the enhancer if CHD4/NuRD(MTA2) was dysfunctional by depleting its essential component CHD4 (Fig. 5g). Our current study suggested that decommission of *TP63* enhancer by TRPS1-CHD4/NuRD(MTA2) in a precision-guided manner contributes to repression of ΔNp63 expression. It is worthy to further investigate how enhancer, P1, and P2 cooperate to mediate the transcription output of *TP63* by TRPS1.

### Silencing TRPS1 increases the metastatic ability of breast cancer cells by increasing ΔNp63 expression

**Fig. 6:**
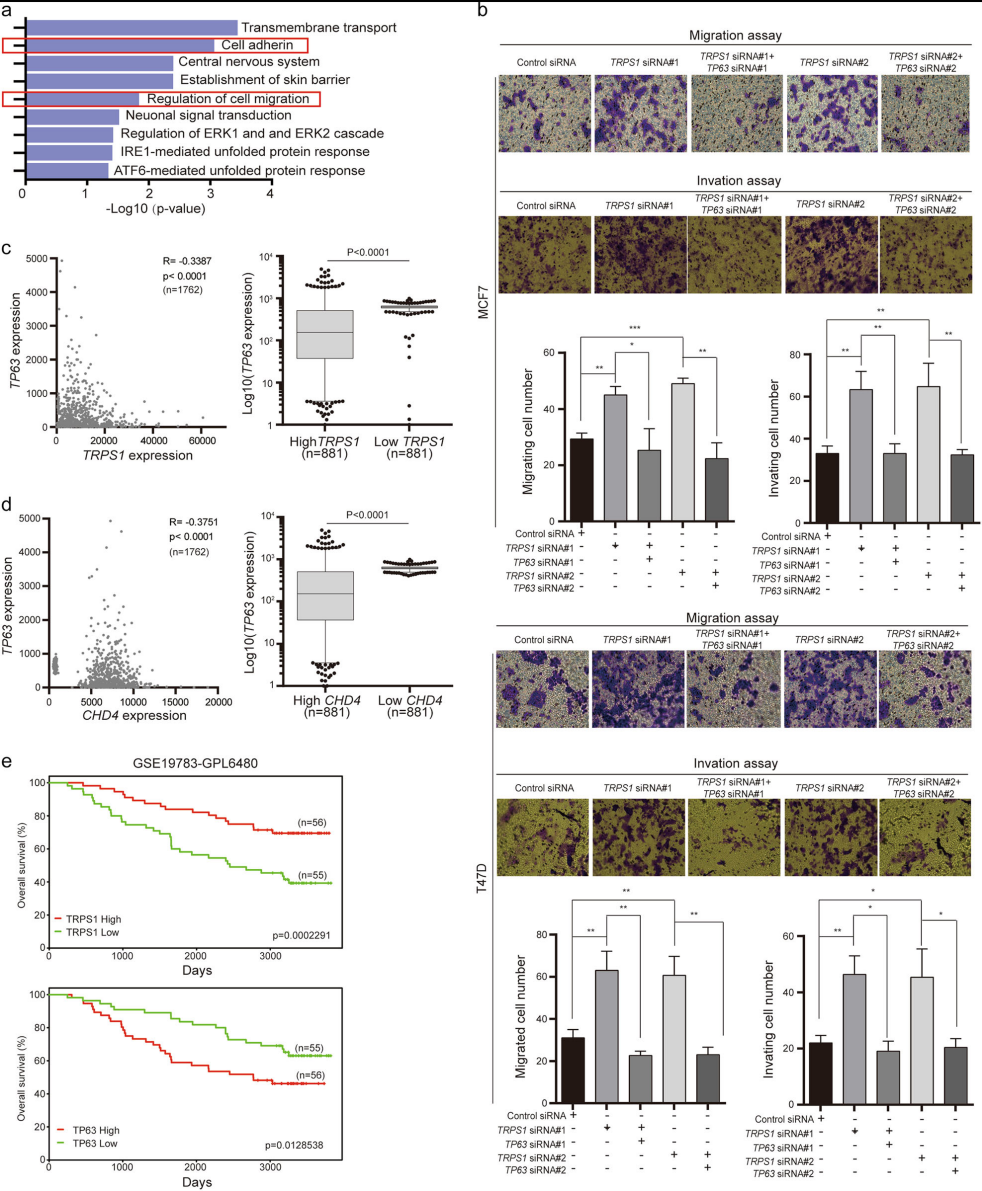
TRPS1-CHD4/NuRD(MTA2)-ΔNp63 axis contributes to breast cancer cell motility and prognosis. **a** Pathway enrichment analysis of TRPS1-targeted genes using DAVID. **b** Migration and invasion assay on T47D and MCF7 cells upon silencing TRPS1 with or without additional silencing of ΔNp63. Each bar represents the mean ± SD for triplicate experiments. *t* Test was used for calculation of statistical significance. *p < 0.05, **p < 0.01, ***p < 0.001. **c** The expression correlation between *TRPS1* and *TP63* using the expression data of 1762 breast cancers from GEO. **d** The expression correlation between *CHD4* and *TP63* using the expression data of 1762 breast cancers from GEO. **c**, **d** *R* stands for correlation and was calculated using GraphPad. *t* Test was used for calculation of statistical significance. **e** Prognostic value of *TRPS1* and *TP63* expression in breast cancers using PROGgene^40^

To better understand the biological functions of TRPS1 associated with its transcriptional repression function in breast cancer pathogenesis, we performed pathway enrichment analysis on the genes directly targeted and transcriptionally repressed by TRPS1 using DAVID^23^ (Fig. 6a). The analysis showed that cell adhesion and regulation of cell migration functions were enriched, suggesting an important role of TRPS1 in regulating metastatic abilities of breast cancer cells. In this context, ΔNp63 was reported to increase cell migration and invasion of breast cancer cells^24,25^. Since ΔNp63 was repressed by TRPS1 (Figs. 4 and 5), we hypothesized that elevated expression of TRPS1 inhibited migration and invasion of breast cancer cells by repressing the expression of ΔNp63. To test this idea, we carried out migration and invasion assays on breast cancer cells with or without silencing *TRPS1*. The number of migrating and invading breast cancer cells increased upon silencing *TRPS1*, while additional depletion of ΔNp63 expression by silencing *TP63* restored the increased migration and invasion abilities of breast cancer cells (Fig. 6b).

We further analyzed the expression data of 1762 breast cancers retrieved from GEO and found a statistically significant negative correlation of the expression between *TRPS1* and *TP63* (*r* = −0.3387, *p* < 0.001) and between CHD4 and *TP63* (*r* = −0.3751, *p* < 0.001) (Fig. 6c, d). Also, breast cancer patients with high TRPS1 and low *TP*63 expression had better overall survival (Fig. 6e). Taken together, our results suggested that elevated expression of TRPS1 nucleates CHD4/NuRD(MTA2) complex to repress ΔNp63 expression to reduce cell migration and invasion of breast cancer cells and better survival.

## Discussion

Herein, we have provided evidence that TRPS1 functions as a transcriptional repressor by recruiting CHD4/NuRD(MTA2) to suppress the expression of target genes. Through physical interaction, TRPS1 and CHD4/NuRD(MTA2) act in an interdependent fashion to execute transcription repression in a precision-guided manner. For example, the expression of ΔNp63 was suppressed by TRPS1-CHD4/NuRD(MTA2), which decommissioned *TP63* enhancer leading to reduced metastatic ability of breast cancer cells and better survival of breast cancer patients. An oncogenic role was ascribed to CHD4 for initiating and supporting tumor-suppressor gene silencing in human colorectal cancer^26^. CHD4 constitutes an essential component of the NuRD complex, which interacts with sequence-specific transcriptional repressors and functions as a multi-protein transcriptional co-repressor^27^. One of the typical members of the GATA family, GATA3, was reported to interact with G9A/NuRD(MTA3) to suppress breast cancer metastasis through targeting the promoters of an array of genes involved in important cellular signaling pathways regulating cell migration and invasion^28^. In our current study, we demonstrated that the atypical GATA member, TRPS1, interacted with CHD4/NuRD(MTA2) to form a transcriptional repressive, precision-guided machinery and targeted both proximal and distal TSS regions of target genes extending the current notion of GATA/NuRD transcriptional repressive function. GATA3 was reported to recruit MTA3 and not MTA2 in MCF7 cells^28^. We showed here that TRPS1 recruited MTA2 in MCF7 cells, indicating differential usage of MTA species specific to different members from the same gene family depending upon the cellular context. In this respect, both GATA3 and TRPS1 were able to regulate multiple genes in the EMT pathway^28^, although the underlying mechanisms are currently not known. Nevertheless, our identification of TRPS1-CHD4/NuRD(MTA2) complex-regulated genes, which are enriched in cell adhesion and cell migration pathways, provides additional support and mechanistic insight into breast cancer metastasis.

The p63 protein, encoded by *TP63*, is a p53 family member involved in a diverse range of biological processes, including embryonic development, cell migration, cell proliferation, differentiation, survival, and apoptosis^29–34^. The role of *TP63* in oncogenesis is controversial due to the complexity of the *TP63* gene, which encodes multiple p63 isoforms^14^, including TAp63 with tumor-suppressive function and ΔNp63 that has an oncogenic role^15,16^. TAp63 and ΔNp63 are the products of two alternative promoters, P1 and P2, respectively, and have pleiotropic functions. Our observation that the TRPS1-CHD4/NuRD(MTA2) complex transcriptionally represses *TP63* via involving decommission of *TP63* enhancer and leading to a reduction of ΔNp63 provided additional mechanistic insight into the transcriptional regulation of *TP63*. Thus the TPRS1-CHD4/NuRD(MTA2) complex is upstream of ΔNp63, which is the main isoform in breast cancer.

In summary, our findings revealed that TRPS1 recruits CHD4/NuRD(MTA2) complex forming precision-guided machinery for the transcriptional repression of specific genes. We have shown that TRPS1 reduced the metastatic ability of breast cancer cells by involving decommission of *TP63* enhancer and repressing ΔNp63 expression. Our data provide mechanistic insights into how TRPS1 functions as a transcription repressor elucidating a new molecular mechanism underlying the transcription regulation of *TP63* and providing a link between TRPS1 and migration and invasion of breast cancer cells. Our results help in understanding the complexity of the functions of GATA family members and support the pursuit of TRPS1, NuRD, and ΔNp63 as the potential prognostic indicators and/or therapeutic targets of breast cancer.

## Materials and methods

### Cell culture

293T cells were cultured in Dulbecco’s modified Eagle’s medium (Life Technologies) supplemented with 10% fetal bovine serum (FBS) and 1% penicillin–streptomycin solution (Life Technologies). MDA-MB-231 cells were maintained in L-15 media with 10% FBS and 1% penicillin–streptomycin solution (Life Technologies). T47D and MCF7 cells were cultured in Roswell Park Memorial Institute medium-1640 (RPMI-1640) (Life Technologies) supplemented with 10% FBS and 1% penicillin–streptomycin solution (Life Technologies).

### RNA interference

Small interfering RNAs (siRNAs) against *TRPS1*, *CHD4*, or *TP63* (coding ΔNp63) and control siRNA (GenePharma) were transfected into cells using Lipofectamine RNAiMAX reagent (Invitrogen). siRNA sequences are listed in Supplementary Table 4.

### Reverse transcription qPCR

Total RNA was extracted using the RNeasy Kit (Qiagen) and reverse transcription of RNA was performed using the PrimeScript RT Reagent Kit (Takara) according to the manufacturers’ instructions. Real-time PCR reactions were performed with SYBR Premix Ex Taq (Takara) in the Bio-Rad CFX96 Real-Time PCR System (Bio-rad). Endogenous β-actin was used for normalization. The primers sequences for real-time RT-PCR are listed in Supplementary Table 4.

### Co-immunoprecipitation

Endogenous Co-IP was performed using anti-TRPS1 antibody (R&D systems), control polyclonal immunoglobulin G (IgG; Santa Cruz Biotechnology, Santa Cruz, CA), and Dynabeads Protein G (Invitrogen, Carlsbad, CA) according to the manufacturers’ instructions. For exogenous Co-IP, we transfected flag-TRPS1 plasmid into 293T cells with PEI. Cells were lysed after 48 h and incubated with flag agarose. Subsequently, western blot analysis of NURD, CHD4, MTA2, HDAC1, HDAC2, and RBBP4/7 was performed.

### GST pull-down assay

Series of TRPS1 truncates were cloned into pET.MBP.3C for generating recombinant proteins. Only TRPS1-∆N (residues 881–1294) could be overexpressed as soluble recombinant protein in *E. coli* DE3 strain. Protein purification was performed according to 6×His-tagged protein purification protocol using Ni-beads following the manufacturer’s protocol (Qiagen).

### ChIP-qPCR

Cells were treated with formaldehyde to create protein–DNA crosslinks, and the crosslinked chromatin was extracted and sheared by sonication. Protein G beads were precleared and blocked with 1% bovine serum albumin. Sheared chromatin was used for immunoprecipitation with normal IgG, TRPS1, or CHD4 antibodies. The immuno-precipitates were washed, eluted, and de-crosslinked. After proteins and RNA were degraded by treatment with proteinase K and RNase A, DNA was purified, followed by quantification PCR. Primer sequences used for ChIP-qPCR experiments are listed in Supplementary Table 4.

### Western blot analysis

CHD4 and his-tag antibodies were acquired from Abcam. The TRPS1 antibody was purchased from R&D Systems. β-Actin antibody and anti-mouse secondary antibody were purchased from Proteintech. The ΔNp63 antibody was bought from Abways Technology. Anti-rabbit secondary antibody and anti-goat secondary antibody were purchased from Santa Cruz Biotechnology.

For western blotting, protein lysates were separated by sodium dodecyl sulfate-polyacrylamide gel electrophoresis, transferred to a polyvinylidene difluoride membrane, and immunoblotted with antibodies. Blots were developed with enhanced chemiluminescence western blotting reagent (Pierce/Thermo Scientific).

### Luciferase reporter constructs

For human *TP63*-minimal CMV-Luc, a 1.6-kb fragment of human *TP63* enhancer coupled with minimal CMV promoter was cloned into the pGL3-Basic plasmid (NheI and HindIII sites) upstream of the firefly luciferase gene (Promega). For mutant human *TP63*-minimal CMV-Luc, conserve sequence WGATAR of human *TP63* enhancer was mutated into WGATCR using the QuikChange II XL Site-Directed Mutagenesis Kit (Agilent Technologies); the corresponding wild-type plasmid was used for mutating A to C following the manufacturer’s procedures.

### Luciferase reporter assay

For the luciferase reporter assay, 293T cells were transfected with *TP63*-minimal CMV-Luc, mutant *TP63*-minimal CMV-Luc, flag-TRPS1, or truncated TRPS1 plasmids containing Renilla luciferase (pRL-SV40). The pRL-SV40 plasmid served as an internal control for normalizing the transfection efficiency. Firefly and Renilla luciferase activities were measured 48 h after transfection with the Dual-Luciferase System (Promega) using a CentroLB960 96-well luminometer (Berthold Technologies).

### Migration and invasion assay

For in vitro migration assay, an 8-μm pore size Boyden chamber (Millipore, PI8P01250) was used. Cells (400 μl, 1 × 10^5^) in 0.5% serum-containing RPMI-1640 were plated in the upper chamber and 600 μl RPMI-1640+10% FBS in the lower chamber as a chemoattractant. For the invasion assay, an 8-μm pore size BD Matrigel Invasion Chamber was used. After 72 h for migration assay and 144 h for invasion assay, cells on the upper side of the filter were removed and cells that remained adherent to the underside of membranes were fixed in formaldehyde, followed by staining with crystal violet. The number of migrated cells was counted using a microscope. Three contiguous fields of each sample were examined using a 20× objective to obtain representative images of cells that migrated/invaded across the membrane.

### Chromatin immunoprecipitation-sequencing and RNA-sequencing

For chromatin immunoprecipitation-sequencing, a total of 5 × 10^7^ T47D cells were used per ChIP assay according to a previously described protocol^35^. Briefly, cells were crosslinked with 1% paraformaldehyde for 10 min at room temperature, quenched with glycine, and fixed chromatin was sonicated and immunoprecipitated with specific antibodies. Libraries were prepared with Illumina’s ChIP-Seq sample prep kit for next-generation sequencing.

For RNA sequencing, T47D cells were transfected with control siRNA, TRPS1 siRNA, or CHD4 shRNA. Seventy-two hours later, total RNA was extracted with the RNeasy Kit (Promega). Total RNA was depleted of rRNA using Ribozero (Illumina), followed by library preparation and next-generation sequencing. The Gene Expression Omnibus (GEO) accession number for the ChIP-seq and expression data reported in this paper is GSE114213.

### Sequencing data analysis

Both the ChIP-seq and RNA-seq data were first trimmed of sequencing adaptors and low sequencing quality end, and the reads passing the quality control were then used for sequence alignment and subsequent analysis. For ChIP-Seq analysis, the reads were aligned to the human reference genome with BWA 0.7.12-r1039. The peaks of ChIP-seq experiment were called from the aligned reads with MACS2 2.1.1.20160309, and the peaks were then annotated for their positions according to the gene structure. The sequences of the peaks were extracted as well for further motif analysis using a python script programmed by our group. The genes with the shortest distance to the peaks were used for enrichment analysis and visualization. To determine TRPS1 motifs, first, the coordinates of the peaks of the TRPS1-binding sites from the ChIP-seq analysis were called with MACS2^36^. Second, DNA-binding motifs over-represented in these peak regions were found using cisFinder, a method by clustering position frequency matrices^37^. All motifs with a false discovery rate <0.05 were defined as TRPS1-binding motifs. After that, the motifs were multiply aligned with clustal Omega, and the aligned motifs were used for calculating the entropy for each position of the certain nucleotide with the method of WebLogo^38^. Finally, the multiplty aligned sequences of nucleotides were filtered with a sliding-window of 10 bp and the entropy value of 2.5. It means that only 10bp continuous nucleotides which has entropy values larger than 2.5 will be kept for logo presentation. For RNA-seq analysis, the reads were aligned with STAR 1.5.2, and the aligned reads were next used to quantify the expression level of the genes with HTSeq 0.9.0. The genes with >2 count per million corresponding to a count of 6–7 reads in sample’s expression were used for the differential expression analysis. A trimmed mean of *M*-values between each pair of samples was performed for normalization. Then the differentially expressed genes were called and used for enrichment analysis and visualization.

## Supplementary information

Supplementary Legends

Figure S1

Figure S2

Supplementary Table 1

Supplementary Table 2

Supplementary Table 3

Supplementary Table 4

Reporting checklist from original submission
