# Supplementary material for: Atypical GATA transcription factor TRPS1 represses gene expression by recruiting CHD4/NuRD(MTA2) and suppresses cell migration and invasion by repressing TP63 expression
Source: Oncogenesis. 2018 Dec 19;7(12):96. doi: 10.1038/s41389-018-0108-9 (PMC6299095; doi:10.1038/s41389-018-0108-9)
Supplement: Supplementary file 1 — Supplementary Legends [file 41389_2018_108_MOESM1_ESM.docx]

**Supplementary Fig. 1** **a** Flowchart for identification interactome of TRPS1. **b** TRPS1 interaction network analysis with STRING database.

**Supplementary Fig. 2** (**a** and **b**) MA plots demonstrating differentially expressed genes after **a** CHD4 or **b** TRPS1 knockdown.

**Supplementary Table 1**: Enrichment of TRPS1 peaks in T47D by Chip-seq.

**Supplementary Table 2** and **3**: Differential expressed genes upon silencing of *TRPS1* or *CHD4* in T47D by RNA-sequencing.

**Supplementary Table 4**: RT-qPCR ,ChIP-qPCR primer sequences and Sequences of siRNAs.
