# Supplementary figures and images for: Atypical GATA transcription factor TRPS1 represses gene expression by recruiting CHD4/NuRD(MTA2) and suppresses cell migration and invasion by repressing TP63 expression
Source: Oncogenesis. 2018 Dec 19;7(12):96. doi: 10.1038/s41389-018-0108-9 (PMC6299095; doi:10.1038/s41389-018-0108-9)

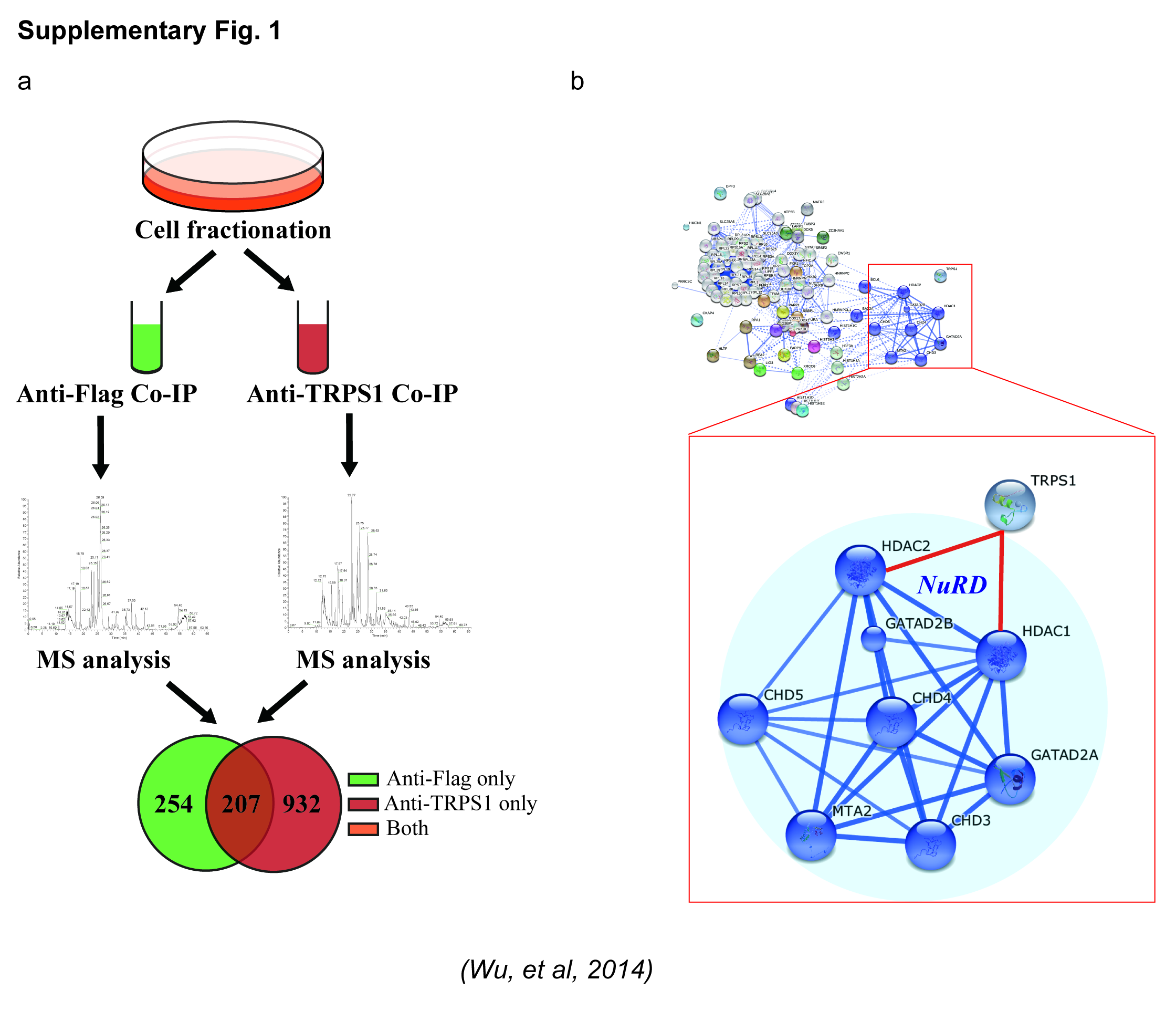

Supplement: Supplementary file 2 — Figure S1 [file 41389_2018_108_MOESM2_ESM.tif]

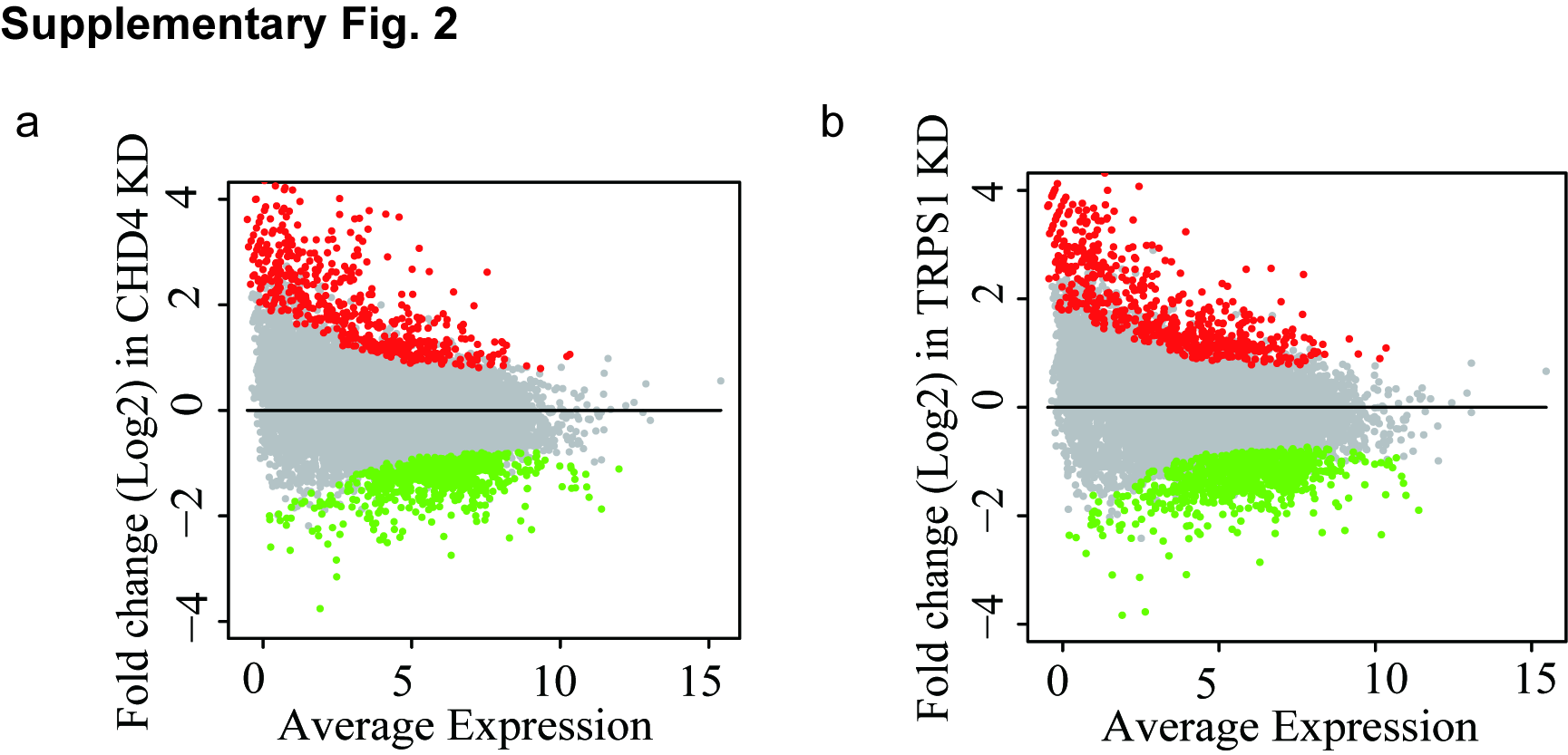

Supplement: Supplementary file 3 — Figure S2 [file 41389_2018_108_MOESM3_ESM.tif]
